# Deceptive beauty of non‐natural structures

**DOI:** 10.1002/pro.70474

**Published:** 2026-01-21

**Authors:** Vsevolod V. Gurevich, Eugenia V. Gurevich

**Affiliations:** ^1^ Department of Pharmacology Vanderbilt University Nashville Tennessee USA

**Keywords:** arrestin, GPCR, protein structure, protein–protein interactions

## Abstract

Structures of proteins and multiprotein complexes are considered landmark achievements. However, in many cases, mutant proteins are used for structural work. Even when wild type proteins are used, crystallization or complex formation for cryoEM is performed in highly nonphysiological conditions. This explains why the structures can be inconsistent with the functional data. The structures are always true, but solved structures faithfully reveal the mode of interactions of the proteins used in the conditions employed. The structures are static, whereas proteins are dynamic. Even when a series of structures are solved, the dynamics are only implied or deduced via molecular dynamics simulations. The mechanisms of protein function in the natural environment can be revealed by the combination of structural, biochemical, biophysical, and in vivo studies, supplemented by molecular modeling.

## 1 INTRODUCTION

Obtaining the structure of a protein or a multiprotein complex of interest is often considered the Holy Grail in biochemistry and cell biology. With the addition of cryoEM to the traditional X‐ray crystallography, the field of cell signaling is getting an avalanche of structures. The structures look beautiful and yield very detailed information that can be used for hypothesis‐driven mutagenesis and for devising molecular tools to manipulate the functions of proteins and their complexes. However, the results of functional studies are not always consistent with structural information. Here we consider the reasons for that, comparing solved structures of arrestin proteins and their complexes with G protein‐coupled receptors (GPCRs) with data obtained by biochemical and biophysical methods as an example.

## 2 FREE ARRESTINS

**FIGURE 1:**
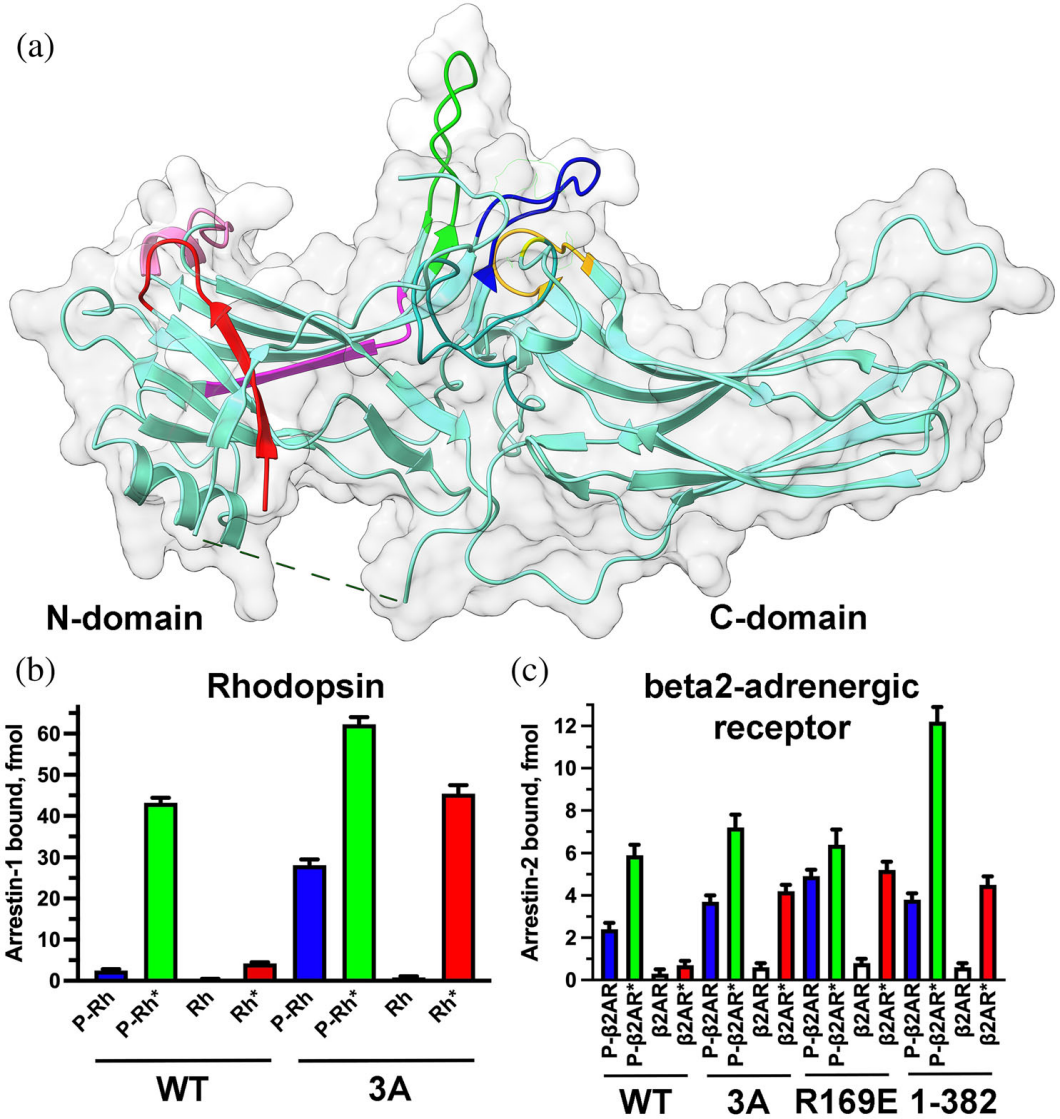
Structure and receptor binding of arrestin proteins. (a) Arrestin‐1 crystal structure (PDB 1CF1) (Hirsch et al., 1999) is shown as light teal flat ribbon with elements studied by comprehensive site‐directed mutagenesis color‐coded, as follows: The N‐terminus (residues 9–21) (Vishnivetskiy et al., 2025a), red; the finger loop (residues 66–79) (Vishnivetskiy et al., 2021), green; β‐strand VI (residues 82–89) (Vishnivetskiy et al., 2023), magenta; the middle loop (residues 132–142) (Vishnivetskiy et al., 2022), blue; N‐edge (residues 156–167) (Vishnivetskiy et al., 2025b), pink; residues 247–254 (Vishnivetskiy et al., 2023), orange; residue 291 (Vishnivetskiy et al., 2023), yellow. Protein surface is shown in pale gray. N‐ and C‐domains are indicated. (b) The effect of 3A mutation (Phe375Ala, Val376Ala, Phe377Ala) in bovine arrestin‐1 on rhodopsin binding. (c) The effects of three activating mutations in bovine arrestin‐2 on its binding to β_2_‐adrenergic receptor: 3A, Arg169Glu, and deletion of the C‐terminus (1–382). The data in panel b are from (Gurevich, 1998), in panel c from (Celver et al., 2002; Kovoor et al., 1999). Panel a was created using UCSF ChimeraX. Panels b and c were created by Prism 10 (Graphpad software, Inc., San Diego, CA). The figure was assembled and labeled in Adobe Photoshop 2025 (Adobe, San Jose, CA).

Vertebrates express four arrestin family members (except bony fish that have undergone the third round of whole genome duplication (Indrischek et al., 2017)): very high levels of arrestin‐1* in rod (Hanson et al., 2007a; Song et al., 2011; Strissel et al., 2006) and cone (Nikonov et al., 2008) photoreceptors in the retina, lower levels of arrestin‐4 in cones (Nikonov et al., 2008), and even lower levels of the two nonvisual subtypes, arrestin‐2 and ‐3 (a.k.a. β‐arrestin1 and 2, respectively) in virtually every cell (reviewed in (Gurevich, 2024)). Full‐length proteins without modifications were used to obtain crystal structures of the basal conformation of all subtypes: arrestin‐1 (Granzin et al., 1998; Hirsch et al., 1999; Sander et al., 2022), arrestin‐2 (Han et al., 2001; Milano et al., 2002), arrestin‐3 (Zhan et al., 2011), and arrestin‐4 (Sutton et al., 2005). In all structures arrestins are elongated molecules consisting of two cup‐like domains, each being a sandwich of two β‐sheets. The only multiturn α‐helix in arrestins is attached to the side of the N‐domain (Figure 1a). The C‐terminus that comes back from the C‐domain and makes contact with the N‐domain is only partially resolved in available structures. In the first published structure of arrestin‐1, likely due to relatively low resolution (3.3 Å), the N‐terminus was erroneously placed where the C‐terminus is localized (Granzin et al., 1998). All other structures revealed two conserved intramolecular interactions that maintain the basal conformation of arrestins. One is the polar core between the two domains consisting of five interacting charged side chains (two arginines and three aspartates) that are virtually solvent‐excluded (Hirsch et al., 1999). The other is a three‐element interaction between β‐strand I and α‐helix of the N‐domain and β‐strand XX of the C‐terminus. Both of these interactions must be broken for arrestin transition into receptor‐bound conformation (Sente et al., 2018). The destabilization of either of these autoinhibitory interactions by mutagenesis greatly increases the propensity of arrestins to bind cognate GPCRs, phosphorylated and unphosphorylated (reviewed in (Gurevich & Gurevich, 2004)). Simultaneous destabilization of both has an even greater effect, apparently reducing the energy barrier of arrestin transition into receptor‐binding conformation (Gurevich et al., 2011).

While the core of all arrestin subtypes looks essentially the same in structures, the positions of the loops connecting β‐strands differ (Han et al., 2001; Hirsch et al., 1999; Milano et al., 2002; Sander et al., 2022; Sutton et al., 2005; Zhan et al., 2011). The loop positions often differ even between protomers of the same arrestin in crystallized oligomers, suggesting that their positions are determined by crystal packing forces, rather than by the arrestin subtype. What all structures show unambiguously is that these loops in arrestins are flexible. This conclusion is consistent with the finding that in some protomers certain loops resolved in others are “invisible.” It is important to remember that what X‐ray crystallography reveals is a 3D electron density. When a flexible protein element “flops” freely the density corresponding to each of many positions it occupies is too low for reliable detection, which makes it invisible (unresolved). This is why in arrestins the C‐terminus (except β‐strand XX anchored by the three‐element interaction) is usually unresolved (Han et al., 2001; Hirsch et al., 1999; Milano et al., 2002; Sander et al., 2022; Sutton et al., 2005; Zhan et al., 2011): it was experimentally shown by NMR to be very flexible (Zhuang et al., 2013). Thus, the X‐ray structures reliably reveal relatively rigid parts of proteins, but do not “see” the most flexible elements. The same applies to cryoEM.

## 3 ARRESTIN‐RECEPTOR COMPLEXES

In contrast to free arrestins, wild type (WT) proteins were never used to obtain the structures of arrestins bound to GPCRs. Mutant forms of both arrestins and receptors were used to increase the stability of the complex. The first structure was that of arrestin‐1 bound to rhodopsin (Kang et al., 2015; Zhou et al., 2017). Inactive WT rhodopsin contains covalently bound inverse agonist 11‐cis‐retinal. Photon of light converts it to covalently bound agonist all‐trans‐retinal, which pushes rhodopsin into active conformation. However, light‐activated WT rhodopsin rapidly loses bound retinal (Hofmann & Lamb, 2023), which precludes its use for structural studies. Therefore, constitutively active mutants are used instead. The solved structure contained mutant human rhodopsin (T4 lysozyme fused at the N‐terminus, two activating mutations Glu113Gln and Met257Tyr, plus Asn2Cys and Asn282Cys to form a stabilizing disulfide bond absent in the WT protein). The protein contained C‐terminally fused mouse arrestin‐1‐(10–392) with triple alanine substitution in the C‐terminus (3A mutation; Leu374Ala, Val375Ala, Phe376Ala in mouse protein) that greatly increases binding to rhodopsin (Gurevich, 1998) (Figure 1b) due to destabilization of the three‐element interaction (Vishnivetskiy et al., 2010). The deletion of the arrestin C‐terminus eliminates β‐strand XX from the three‐element interaction, thereby similarly increasing the binding to receptors, most dramatically to their nonpreferred by arrestins unphosphorylated form (Celver et al., 2002; Gurevich, 1998; Kovoor et al., 1999; Zheng et al., 2023). The replacement of the positively charged arginine in the polar core with negatively charged glutamate (Arg175Glu and Arg169Glu in arrestin‐1 and ‐2, respectively) has the same effect (Celver et al., 2002; Gurevich, 1998; Kovoor et al., 1999) (Figure 1c).

**FIGURE 2:**
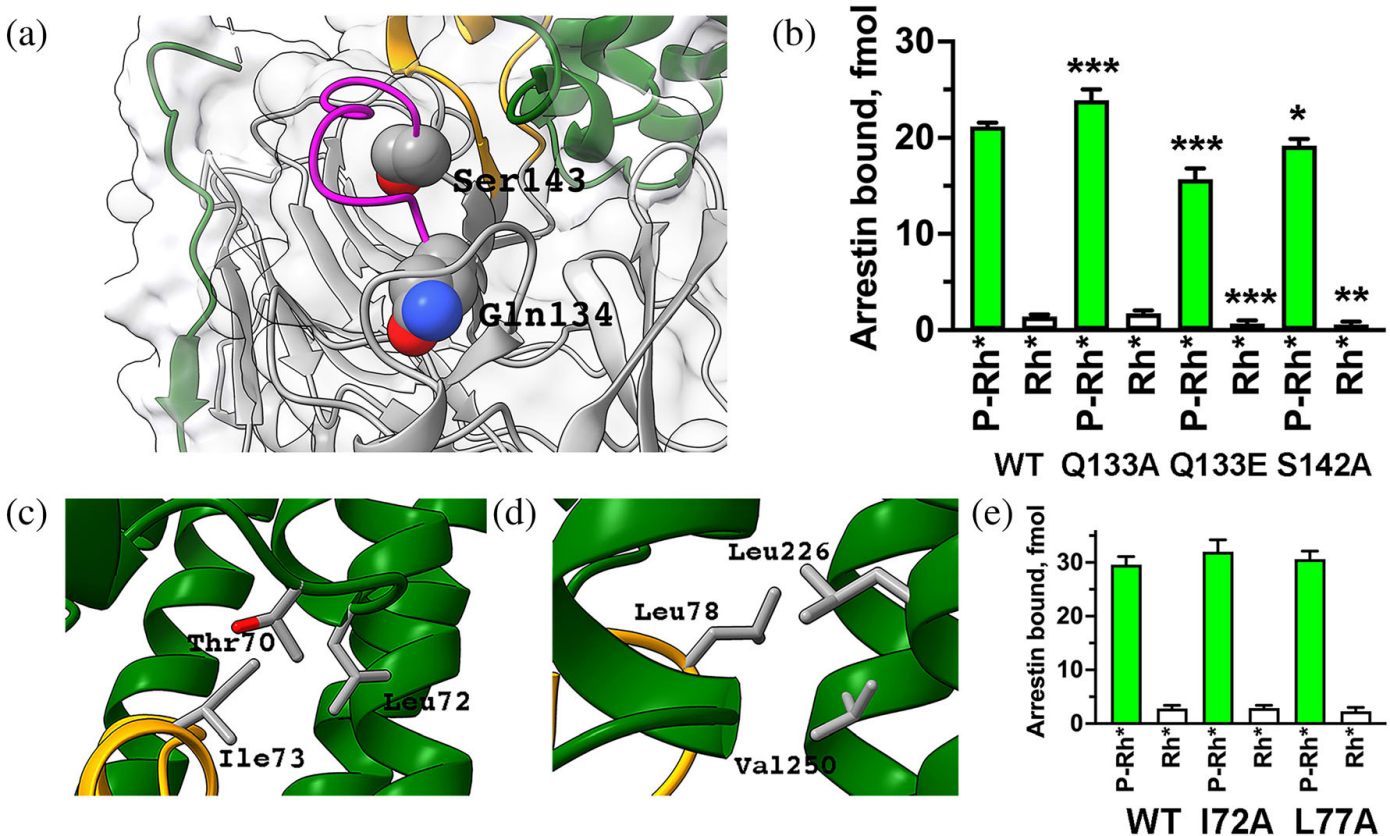
Structure–function comparisons in arrestin‐1. (a) The structure of the arrestin‐1 bound to rhodopsin (Kang et al., 2015) with Gln134 and Ser143 shown as CPK models. Mouse arrestin‐1 is shown in gray, its finger loop in yellow, middle loop in magenta, rhodopsin in green. (b) The effects of substitutions of homologous Gln133 and Ser142 in bovine arrestin‐1. Bars representing the binding to light‐activated phosphorylated rhodopsin (P‐Rh*) are green, representing the binding to light‐activated unphosphorylated rhodopsin (Rh*) are white. (c, d) Mouse arrestin‐1 Ile73 (c) and Leu78 (d) and nearby rhodopsin residues in the structure of the complex (Kang et al., 2015) are shown as stick models. Arrestin‐1 and rhodopsin are shown in yellow and green, respectively. (e). Alanine substitution of homologous Ile72 and Leu77 in bovine arrestin‐1 did not affect rhodopsin binding. Statistical significance of the differences between WT arrestin‐1 and mutants was determined by one‐way ANOVA followed by Dunnet post hoc test with correction for multiple comparisons and indicated, as follows: *p < 0.05; **p < 0.01; ***p < 0.001 to WT. Panels a, c, and d were generated using UCSF ChimeraX, panels b and e by Prism 10 (Graphpad software, Inc., San Diego, CA). The figure was assembled in Adobe Photoshop 2025 (Adobe, San Jose, CA).

Rhodopsin is the only GPCR that is relatively easy to purify in large quantities (e.g., from cow eyes (Hargrave, 1976)) in unphosphorylated and phosphorylated by endogenous rhodopsin kinase (systematic name GRK1) form (McDowell et al., 1993)). Cell‐free translation produces radiolabeled fully functional arrestin‐1 (Gurevich & Benovic, 1992). The availability of these tools enabled the development of a direct binding assay with femtomolar sensitivity (Gurevich & Benovic, 1992), which was used for numerous structure–function studies. Recent studies compared the effects of point mutations in several elements of bovine arrestin‐1 on the binding of WT protein and its “enhanced” mutants (3A or 1–378 with deleted C‐terminus) to the preferred target of arrestin‐1, light‐activated phosphorylated rhodopsin, as well as to its light‐activated unphosphorylated form. A total of 107 mutations in bovine arrestin‐1 were tested (Figure 1a). The effects of two thirds of these (72 out of 107) in different parts of the molecule on the background of WT arrestin‐1 and its enhanced mutants were different. Predictions based on the solved structure of the complex (Kang et al., 2015; Zhou et al., 2017) were not always supported by the functional data obtained with WT proteins. Here are just two examples. Gln134 and Ser143 in mouse arrestin‐1 in the structure are localized far from bound rhodopsin (Figure 2a) (Kang et al., 2015). Yet mutations of homologous Gln133 and Ser142 in the bovine arrestin‐1 (because of an extra residue in the N‐terminus, which does not participate in receptor binding, mouse numbers are N + 1 compared to bovine) significantly affected the binding (Figure 2b) (Vishnivetskiy et al., 2022). In contrast, Ile73 and Leu78 in mouse arrestin‐1 are within interaction distance from several rhodopsin residues in the complex (Figure 2c,d) (Kang et al., 2015). However, alanine substitutions of homologous bovine Ile72 and Leu77, which replaced their long side chains with a very short one, precluding possible interactions, did not affect rhodopsin binding (Figure 2e) (Vishnivetskiy et al., 2021). The most parsimonious explanation is that the prevalent shape of the complex of WT arrestin‐1 with WT rhodopsin is different from the shape of the complex of the two mutants revealed by the structure.

The structures of arrestin‐2 bound to several GPCRs (Bous et al., 2022; Cai et al., 2025; Cao et al., 2022; Chen et al., 2023; Chen et al., 2025; Huang et al., 2020; Lee et al., 2020; Liao et al., 2023; Sun et al., 2025; Thomsen et al., 2016; Yin et al., 2019) and two of arrestin‐3 bound to atypical chemokine receptor ACKR3 (Chen et al., 2025) and to GPR1 (chemerin receptor) (Cai et al., 2025) have been solved. Mutant forms of both GPCRs and arrestins were always used to obtain stable complexes (Table 1). Mutations in GPCRs can dramatically affect their function. For example, neurotensin NTSR1 was thermostabilized by mutagenesis in the agonist‐binding conformation, but this mutant failed to couple to G protein (Shibata et al., 2009). As a rule, functional capabilities of the mutant receptors used for these structural studies have not been tested. Case‐by‐case details are given in Table 1, so here we only describe the complex of arrestin‐2 with M2 muscarinic receptor (M2R). Arrestin‐2 binding to WT M2R requires receptor phosphorylation (Gurevich et al., 1995). M2R has no phosphorylation sites in its very short C‐terminus; all phosphorylation sites important for arrestin binding are localized in the large third cytoplasmic loop (ICL3) (Lee et al., 2000). Yet to obtain this structure, the ICL3 was deleted, and native C‐terminus was replaced using sortase with multiphosphorylated C‐terminal peptide of V2 vasopressin receptor (V2Rpp) (Staus et al., 2020), which was previously shown to tightly bind arrestin‐2 (Shukla et al., 2013).

**TABLE 1:** Arrestin complexes with GPCRs.

Receptor mutations	Arrestin‐2 mutations	Additional information	Reference
NTSR1 neurotensin receptor (residues 49‐418) with BRIL (engineered four‐helix bundle protein derived from E. coli cytochrome b562) fused to the N‐terminus	Arrestin‐2‐(1‐393) with 3A mutation in the C‐terminus	NTSR1, arrestin‐2, and light chain of Fab30 were fused	Yin et al. (2019)
NTSR1 with N‐terminal Flag plus His8 tags	Cysteine‐less (seven mutations) arrestin‐2‐(1‐382)	The complex was cross‐linked	Huang et al. (2020)
NTSR1 with N‐terminal BRIL	Arrestin‐2‐(1‐382)	Fab30 added	Sun et al. (2025)
β1‐adrenergic receptor with 32 residues of the N‐terminus and part of the ICL3 (from Cys244 to Arg271) deleted, nine stabilizing point mutations, thioredoxin fused to the N‐terminus, native C‐terminus downstream of residue 358 replaced with V2Rpp	Arrestin‐2 with Leu68Cys mutation in the finger loop, Arg169Glu mutation, and Flag tag on the C‐terminus	Fv30 antibody was added	Lee et al. (2020)
5HT_2B_ serotonin receptor with large deletion in ICL3 (Ala248‐Val313), mutations Lys247Val and Glu319Leu, BRIL fused to the N‐terminus, native C‐terminus replaced with V2Rpp	Arrestin‐2‐(1‐368) with Arg169Glu mutation	Receptor, arrestin‐2, and ScFv30 antibody were fused	Cao et al. (2022)
V2 vasopressin receptor with N‐terminal Flag tag and Asn22Gln mutation	Arrestin‐2‐(1‐382)	Fv30 antibody was added	Bous et al. (2022)
Cannabinoid CB1 receptor with C‐terminus (after residue 413) replaced with V2Rpp	Arrestin‐2‐(1‐393) with peptide 86 fused to its N‐terminus and two activating mutations, Arg169Glu and 3A	Fab30 was added	Liao et al. (2023)
M2 muscarinic receptor with N‐terminal Flag tag, deleted large ICL3, and C‐terminus replaced with V2Rpp	Cysteine‐less (seven mutations) arrestin‐2‐(1‐393) with N‐terminal MBP	Fv30 or Fv30 plus Nb24 were added	Staus et al. (2020)
Glucagon GCGR with C‐terminus replaced with V2Rpp	Arrestin‐2‐(1‐376)	Fv30 was fused to the C‐terminus of arrestin‐2	Chen et al. (2023)
β2‐adrenergic receptor (1–341) with N‐terminally fused T4 lysozyme and native C‐terminus replaced by the 29‐residue C‐terminus of V2 receptor	Arrestin‐2‐(1‐393)	Fab30, Nb32, and Nb35 were added	Nguyen et al. (2019), Thomsen et al. (2016)
Atypical chemokine receptor ACKR3 (2–362) with N‐terminal HA tag and C‐terminal 10xHis and Flag tags	Arrestin‐2‐3A and arrestin‐3‐(1–392)	Fab7 was added	Chen et al. (2025)
GPR1 (chemerin) receptor with the C‐terminus (Leu323 to Gln355) replaced by V2 receptor C‐terminus (Ala343 to Ser371). Inactive GPR1 receptor also carried Val143Cys mutation to make a disulfide bond with arrestin‐2‐Tyr249Cys.	Cysteine‐less (7 mutations) arrestin‐2‐Arg169Glu with fused scFv30. To obtain the structure with inactive receptor arrestin‐2 contained an additional mutation Tyr249Cys.	Nb32 was added	Cai et al. (2025)
GPR1 (chemerin) receptor with the C‐terminus (Leu323 to Gln355) replaced by V2 receptor C‐terminus (Ala343 to Ser371).	Cysteine‐less (eight mutations) arrestin‐3 with the C‐terminus (Asp377 to Cys409) replaced with antibody scFv30 and 6xHis tag. Sequence from His350 to Phe376 was replaced with arrestin‐2 sequence from Lys357 to Leu376 to make scFv30 bind it.	Nb32 was added	Cai et al. (2025)

In addition to containing mutant receptors and mutant forms of arrestin‐2 and ‐3 (Table 1), the complexes were stabilized by fusing the receptor and arrestin‐2 (Cao et al., 2022; Yin et al., 2019), by conformation‐specific antibodies (Bous et al., 2022; Cai et al., 2025; Cao et al., 2022; Chen et al., 2023; Chen et al., 2025; Lee et al., 2020; Liao et al., 2023; Nguyen et al., 2019; Staus et al., 2020; Sun et al., 2025; Yin et al., 2019), and in one case by cross‐linking (Huang et al., 2020). Two structures revealed “tail” engagement of arrestin‐2 (Chen et al., 2023; Thomsen et al., 2016). However, in both cases the “tail” was V2Rpp, not the native C‐terminus of the β2‐adrenergic (Thomsen et al., 2016) or glucagon (Chen et al., 2023) receptor. The receptor binding of arrestin‐2 mutants with destabilized polar core or three‐element interaction used for structure determination is dramatically different from that of WT arrestin‐2 (Figure 1c). While receptor binding of some of the mutants used in structural studies was not compared to WT in direct binding assay with purified WT receptors, the data obtained with arrestin‐1 and rhodopsin (Vishnivetskiy et al., 2021; Vishnivetskiy et al., 2022; Vishnivetskiy et al., 2023; Vishnivetskiy et al., 2025a; Vishnivetskiy et al., 2025b) give one pause.

Another important aspect is that GPCRs in vivo reside in lipid bilayer. Only three structures of the receptor‐arrestin complexes were determined in nanodiscs mimicking relatively flat biological membranes (Chen et al., 2025; Lee et al., 2020; Staus et al., 2020), whereas in most cases the receptor was in small detergent micelles with significant curvature (Cai et al., 2025; Cao et al., 2022; Chen et al., 2023; Huang et al., 2020; Kang et al., 2015; Liao et al., 2023; Nguyen et al., 2019; Sun et al., 2025; Thomsen et al., 2016; Yin et al., 2019; Zhou et al., 2017). The tilt of receptor‐bound arrestin is often discussed, although it stands to reason that it was significantly affected by the nature of the receptor‐containing entity in solved structures (Aydin et al., 2023).

The software for structure prediction is rapidly improving. However, even arguably the highest current achievement, Alphafold3, suffers from “hallucinations” (predicting “structure” of intrinsically disordered protein elements) and sometimes produces structures of complexes with clashes between constituent molecules, as its developers acknowledged (Abramson et al., 2024). Conceptually, the inherent weakness of structure‐predicting software is that it is inevitably “trained” on experimental structures, which can contain artifacts because of the use of mutant proteins and/or nonphysiological conditions. Such software can be tested by functional characterization of mutants where residues predicted to be critical for function, folding, or interaction with a partner are changed. This rigorous experimental testing remains to be performed.

## 4 THE CASE OF ARRESTIN OLIGOMERS

**FIGURE 3:**
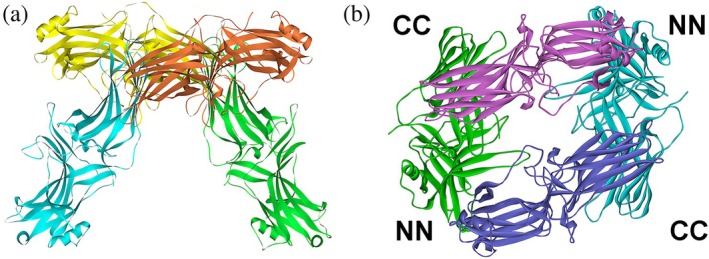
Arrestin‐1 forms different tetramers in crystal and in solution. (a) Crystal tetramer of bovine arrestin‐1 (PDB 1CF1) (Hirsch et al., 1999)). (b) A model of solution tetramer confirmed by site‐directed mutagenesis (Hanson et al., 2008a). Protomers in both panels are shown in different colors for clarity. The interactions between N‐ (NN) and C‐ (CC) domains of the protomers in the solution tetramer are indicated in panel b. Panels were generated using UCSF ChimeraX. The figure was assembled and labeled in Adobe Photoshop 2025 (Adobe, San Jose, CA).

WT arrestins were crystallized in the basal state (Granzin et al., 1998; Han et al., 2001; Hirsch et al., 1999; Milano et al., 2002; Sander et al., 2022; Sutton et al., 2005; Zhan et al., 2011). The structures of the protomers in crystal oligomers of all arrestin subtypes are remarkably similar and satisfyingly consistent with the functional data. Notably, solved structures of the two preactivated “enhanced” forms of arrestin‐1, C‐terminally truncated p44 (Kim et al., 2013) (equivalent of C‐terminally truncated forms of arrestin‐2) and Arg175Glu (equivalent of arrestin‐2‐Arg169Glu) polar core mutant (Granzin et al., 2015) were quite different from those of full‐length WT protein (Hirsch et al., 1999; Sander et al., 2022). Three labs, one in Germany (Granzin et al., 1998) and two in the United States (Hirsch et al., 1999; Sander et al., 2022), in different crystallization conditions obtained virtually identical bovine arrestin‐1 tetramers (Figure 3a). As arrestin‐1 was shown to oligomerize forming dimers and tetramers (dimers of dimers) in solution (Schubert et al., 1999), taken at face value, these results suggested that the structures revealed the shape of the biological tetramer. To test this hypothesis, single spin labels were introduced at different positions of purified arrestin‐1. It was concentrated to make it tetramerize, and distances between protomers in the tetramer were measured by pulse electron paramagnetic resonance technique double electron–electron resonance (DEER) (Hanson et al., 2007b). Surprisingly, none of the experimentally measured distances matched those predicted by the crystal tetramer (Hanson et al., 2007b). Modeling based on numerous protomer‐protomer distances measured by DEER suggested a totally different protomer arrangement in bovine arrestin‐1 tetramer (Hanson et al., 2008a) (Figure 3b). In this model phenylalanines in positions 85 and 197, which do not mediate protomer‐protomer interactions in the crystal tetramer (Granzin et al., 1998; Hirsch et al., 1999; Sander et al., 2022), appeared to play critical role in the interactions between protomers (Hanson et al., 2008a). Alanine substitutions of these two phenylalanines suppressed oligomerization of bovine arrestin‐1, consistent with the solution tetramer model (Hanson et al., 2008a). Homologous mutations similarly suppressed oligomerization of mouse arrestin‐1 (Kim et al., 2011), suggesting that the mechanism of its self‐association is conserved in mammals. Thus, the shape of the solution tetramer of arrestin‐1 is dramatically different from that observed in crystals. Intracellular concentration of arrestin‐1 in rods is ~2 mM (Song et al., 2011). Measured dimerization and tetramerization constants of bovine and mouse arrestin‐1 are below 65 μM (Hanson et al., 2008b; Kim et al., 2011), which means that the bulk of arrestin‐1 in photoreceptors exists in oligomeric form. Experiments in genetically modified mice suggest that solution tetramer predicted based on DEER distance measurements is biologically relevant, reflecting the molecular mechanism of arrestin‐1 self‐association in vivo (Samaranayake et al., 2020).

Both nonvisual arrestin‐2 and ‐3 also oligomerize (Hanson et al., 2008b; Milano et al., 2006; Scott et al., 2002). In contrast to arrestin‐1, an abundant cytoplasmic metabolite inositol‐hexa‐*kis*‐phosphate (IP_6_) facilitates their self‐association (Hanson et al., 2008b). IP_6_‐soaked crystals of WT arrestin‐2 (Milano et al., 2006) revealed “infinite” chains with protomers in basal conformation. Interprotomer distances in arrestin‐2 oligomers formed in the presence of IP_6_ in solution were measured by DEER and found to match those predicted by the structure (Chen et al., 2021). Also in agreement with the structure (Milano et al., 2006), distance measurements showed that the C‐termini of protomers are in their basal position, attached to the N‐domain (Chen et al., 2021). Intracellular concentrations of endogenous arrestin‐1 in rods are four orders of magnitude higher than that of nonvisual arrestins in other cells (Gurevich et al., 2004; Song et al., 2011). Measured dimerization and tetramerization constants compared with the level of arrestin‐1 expression suggest that the majority of arrestin‐1 exists in rods in the form of dimers and tetramers (Kim et al., 2011). In contrast, the concentration of endogenous arrestin‐2 is ~100 nM in highly expressing neurons (Gurevich et al., 2004). But it self‐associated at much higher concentrations, above 1 μM (Hanson et al., 2008b). Arrestin‐3‐(1‐393) in the presence of IP_6_ crystallizes as a trimer, in which all protomers are in receptor bound‐like conformation (Chen et al., 2017). The intracellular concentrations of endogenous arrestin‐3 are even lower than those of arrestin‐2 (<30 nM) (Gurevich et al., 2004). Although full‐length arrestin‐3 oligomerizes (Hanson et al., 2008b), it has not been shown to form the same trimers as the truncated (1‐393) mutant. Therefore, it is unclear whether at least some arrestin‐3 exists in oligomeric form in the cytoplasm of living cells.

Thus, structural data regarding oligomers were shown to be wrong and correct in case of arrestin‐1 and arrestin‐2, respectively. It is impossible to know which is the case for arrestin‐3 without testing structural predictions by other methods.

## 5 DYNAMICS OF PROTEINS AND MULTIPROTEIN COMPLEXES

Structures are inherently static, whereas proteins are highly dynamic molecules. This has been experimentally demonstrated in the case of several GPCRs and arrestins by biophysical methods. Rhodopsin (Elgeti & Hubbell, 2021), β_2_‐adrenergic (Manglik et al., 2015), and M2R (Xu et al., 2019) were shown to exist in a multiconformational equilibrium. Ligands and interacting proteins shifted this equilibrium but never forced all receptor molecules into a single conformation (Elgeti & Hubbell, 2021; Manglik et al., 2015; Xu et al., 2019). Both free (Carter et al., 2005; Hanson et al., 2006; Zhuang et al., 2013) and receptor‐bound arrestins (Asher et al., 2022; Hanson et al., 2006; Zhuang et al., 2013; Zhuo et al., 2014) also sample multiple conformations. Thus, the evidence that the complex of the same arrestin with the same receptor exists in many different shapes was hardly surprising. DEER measurements between selected residues in rhodopsin and bound arrestin‐1 invariably yielded several distances with each pair (Kang et al., 2015; Zhou et al., 2017). While the most probable distances matched solved structures, the others demonstrated coexistence of the forms of the complex that were not captured in crystal. The same conclusion was reached in the investigation of arrestin‐2 interactions with parathyroid hormone receptor 1 in living cells (Aydin et al., 2023). In that study, cysteine residues were inserted at different places on the cytoplasmic surface of the receptor and cysteine‐reactive unnatural amino acids were inserted at different places on the receptor‐binding side of arrestin‐2. The experiments identified 136 proximity points between the receptor and arrestin‐2. Molecular dynamics (MD) simulations showed that no single conformation can account for all of these proximity points, but the entire spectrum of possible conformations can (Aydin et al., 2023). This natural heterogeneity is anathema for the structural studies.

When sufficient number of particles is available for analysis (hundreds of thousands selected from millions acquired), cryoEM allows simultaneous structure determination of several different “flavors” of the complex (Cai et al., 2025). Using these enormous datasets, four distinct conformations of GPR1‐arrestin‐2 complex were resolved (Cai et al., 2025). Because of different tilt of the arrestin‐2 molecule relative to the receptor, it was hypothesized that these complexes reveal the stages of arrestin‐2 interaction with this GPCR (Cai et al., 2025). However, the structure of only one type of arrestin‐3‐GPR1 complex was solved (Cai et al., 2025). In this complex the long axis of arrestin‐3 is virtually parallel to the micelle surface, similar to arrestin‐2 complexes hypothesized to represent an early stage of the binding process (Cai et al., 2025).

The process of G_s_ activation by β2AR upon GTP addition was analyzed using time‐resolved cryoEM, that is, sequential freeze‐trapping at 5, 10, and 17 seconds after GTP addition to the preformed β2AR‐G_s_ complex (Papasergi‐Scott et al., 2024). The authors took advantage of the fact that the process of G_s_ activation is significantly slowed down when the receptor is in detergent micelle, as compared to its subsecond rate with the receptor in the membrane (Gregorio et al., 2017). The analysis of millions of particles combined with extensive MD simulations yielded a plausible reconstruction of the molecular events in the process of G_s_ activation, from GTP binding to the dissociation of the activated GTP‐liganded Gα‐subunit from the receptor and Gβγ (Papasergi‐Scott et al., 2024). Undoubtedly, time‐resolved cryoEM is a significant breakthrough. However, in this study, the information extracted from a series of structures was greatly increased by MD simulations. The finding that in terms of structural rearrangements in the process of activation and subsequent dissociation from the receptor Gα largely retraces the steps it followed during initial binding to a GPCR (Papasergi‐Scott et al., 2024) is reassuring, as it makes perfect sense from a biochemical standpoint.

## 6 STRUCTURE IS A STEP FORWARD

In their natural environment, proteins sample a wide range of conformations. Most biologically relevant multiprotein complexes are transient and structurally heterogenous. Does this mean that structural studies, that can be successful only with stable, rigid, and homogenous proteins and complexes, are inherently misleading? Of course not. Obviously, X‐ray crystallography and cryoEM have their limitations. Proteins were not “designed” by evolution to form crystals, so to obtain the structure they are forced to crystallize by extreme from a biological standpoint conditions: high ionic strength, molecules that take water away from them, such as polyethylene glycol, etc. Very often the most dynamic elements, which are usually functionally important, are chopped off to obtain the structure of the remaining part. Protein–protein complexes that regulate cellular signaling are always transient, as the cell is a very dynamic system. Therefore, for structural studies, these complexes are stabilized by artificial means: mutations, fusions, antibodies, cross‐linking, etc. Solved structures reflect these limitations. However, they yield detailed information that cannot be obtained by other methods. Structures are very useful guides for functional experiments, although the results of these might contradict structure‐based predictions. In these cases, functional data appear to be more trustworthy. To get the most biologically relevant information out of structures, we need to keep in mind that each solved structure is an important step on the way, not the final destination. We need to know what the proteins and their complexes look like in the cell, where they function. This requires a wealth of structure–function information obtained by biochemical and biophysical methods. The results of cell biological, biochemical, and biophysical experiments can also be misleading. So, scientists need to be equally careful with structural and functional data, always separating grain from the chaff by combining different approaches. As proteins and their complexes contain more elements than the human mind can handle simultaneously, the synthesis of structural information with the functional data can be achieved by molecular modeling.

## AUTHOR CONTRIBUTIONS

**Eugenia V. Gurevich:** Writing – original draft; writing – review and editing. **Vsevolod V. Gurevich:** Conceptualization; funding acquisition; visualization; writing – original draft; writing – review and editing; supervision.

## Data Availability

The data that support the findings of this study are openly available in PubMed at https://pubmed.ncbi.nlm.nih.gov.

## Footnotes

* We use systematic names of arrestin proteins. The number after the dash indicates the order of cloning: arrestin‐1 (historic names S‐antigen, 48 kDa protein, visual or rod arrestin; SAG in HUGO database), arrestin‐2 (β‐arrestin or β‐arrestin1; ARRB1 in HUGO database), arrestin‐3 (β‐arrestin2 or hTHY‐ARRX; ARRB2 in HUGO database), and arrestin‐4 (cone or X‐arrestin; ARR3 in HUGO database).
